# Biological Impact of Photoperiod on Fairy Shrimp (*Branchinecta orientalis*): Life History and Biochemical Composition

**DOI:** 10.3390/biology10080695

**Published:** 2021-07-22

**Authors:** Sara Farhadi, Behrooz Atashbar Kangarloei, Ahmad Imani, Kourosh Sarvi Moghanlou

**Affiliations:** 1 Department of Fisheries, Faculty of Agriculture and Natural Resources, Urmia University, Urmia 57561-51818, Iran; st_s.farhadi@urmia.ac.ir (S.F.); a.imani@urmia.ac.ir (A.I.); k.sarvimoghanlou@urmia.ac.ir (K.S.M.); 2 Department of Ecology and Stocks Management, Artemia and Aquaculture Research Institute, Urmia University, Urmia 57179-44514, Iran

**Keywords:** *B. orientalis*, photoperiod, growth, fatty acid, amino acid, digestive enzyme

## Abstract

**Simple Summary:**

*Branchinecta orientalis* G.O. Sars, 1901 is a broadly distributed fairy shrimp species in temporary freshwater pools throughout Europe and Asia. Recently, using fairy shrimps to feed freshwater fish and shellfish species has been brought to attention mainly due to their high nutritional value, possibility of mass culture, and ability to remain alive for long periods when used as prey. Fairy shrimps might be valuable alternatives for the widely used brine shrimp *Artemia* species; however, relatively little is known regarding their life-cycle characteristics and biochemical properties under various environmental conditions. Among environmental factors, the photoperiod is assumed as an important environmental cue to regulate the growth, development, and physio-biochemical properties of animals. In the present study, the growth performances, reproductive status, and nutritional quality of fairy shrimp were investigated under predefined environmental conditions, i.e., different photoperiods, and compared with various common live prey used in freshwater ornamental fish production.

**Abstract:**

*B. orientalis*, fairy shrimp, is often among the most conspicuous invertebrates inhabiting temporary aquatic habitats with a typical variation in environmental conditions. Its life history characteristics and biochemical composition were studied under four different photoperiodic regimes (24L:0D, 0L:24D, 16L:8D, and 12L:12D). The significantly highest cumulative and initial hatching rates (48 h) were obtained at 24L:0D (*p* < 0.05). Cultivating the larvae under different photoperiods did not significantly affect specific growth rate (SGR) (*p* > 0.05). However, higher final total body length and daily growth rate were recorded under constant darkness. Higher lipid content was found at 24L:0D to the extent that it was more than two times higher than that at 16L:8D and 12L:12D (*p* < 0.05). There was also a remarkable increase in body crude protein content at 24L:0D (*p* < 0.05). Body fatty-acid profiles of the fairy shrimps were also affected by culture condition (*p* < 0.05). Extension of lighting period resulted in a subtle increase in body contents of arginine, lysine, histidine, isoleucine, leucine, valine, methionine, and phenylalanine, especially in the group kept under a 16L:8D regime. The highest and lowest digestive enzyme activity was observed at 0L:24D and 24L:0D, respectively (*p* < 0.05). In contrast, the highest and lowest soluble protein content was recorded at 24L:0D and 0L:24D, respectively (*p* < 0.05). Similarly, antioxidant status was significantly higher at 0L:24D (*p* < 0.05). In conclusion, a 16L:8D light–dark cycle might be an optimal condition in terms of growth performance and physio-biochemical characteristics. These findings could be helpful in optimizing the rearing conditions for upscaling *B. orientalis* production.

## 1. Introduction

Anostracans (Crustacea, Branchiopoda), known as fairy shrimps, are often the dominant fauna of temporary freshwater pools [1]. They have mostly adapted to a specific set of environmental parameters such as temperature, salinity, and pH [2]. *B. orientalis* G. O. Sars, 1901 is a widely distributed species of fairy shrimp, extending from Mongolia to Austria [3,4]. In terms of topology, it has a diverse distribution in lowland or high-altitude temporary pools [5]. *B. orientalis* was recently reported as one of the typical large Branchiopoda inhabitants in West Azerbaijan, Iran [6].

Several researchers have studied the population dynamics of fairy shrimps under varying environmental conditions (e.g., [7,8]). Meanwhile, reports on the relationship between environmental factors (e.g., temperature and food) and the life history characteristics (e.g., hatching rate, growth performance, average lifespan, survivorship, and fecundity) of *B. orientalis* are scarce [9,10].

The photoperiod is assumed as an important environmental cue to regulate the growth, development, maturity, and other vital physiological traits of animals [11]; however, it should be noted that other ambient factors including temperature, lunar cycle, pH, population density, and food availability might also play an important role in this regard. In aquatic species, it has been successfully used to improve growth during different life stages [12,13,14,15]. As an external stimulus, photoperiod triggers many physio-biochemical processes, such as tissue growth and circadian rhythms of development and enzymes activity, in most crustaceans [16]. In Artemia, digestive systems also mainly develop and function during the early life stage, and their ontogenetic development and functioning directly determine the survival, growth, and nutritional condition [17]. It has been suggested that the photoperiod is one of the most important factors explaining the seasonal pattern of emergence detected for many temperate zooplankton populations [18,19]. Moreover, it may be a more reliable signal for the synchronization of life-cycle phases to seasonal cycles in the habitats [20].

Light-induced hatching has been widely reported in branchiopod crustaceans, with different light conditions (e.g., in terms of light intensity and photoperiod), resulting in different hatching success [21,22]. The photoperiod usually plays a key role in inducing diapause in different groups of crustaceans including copepods, cladocerans, and amphipods [23], as well as different species of Artemia [24].

It is assumed that the introduction and provision of a reliable source of live food, especially during the early life stage of various aquaculture organisms, has considerably improved aquaculture success during the last decade [25]. Most fairy shrimps might grow from about 0.5 to 10–15 mm in 14 days. Such a wide size range would make it a suitable choice for feeding all sizes of freshwater ornamental fish species up to 10 cm in length, including juvenile discus, *Symphysodon* spp., guppy, and *Poecilia reticulata*. Such live prey might also be applicable regarding feeding of brooders [26]. Live feed is preferred over formulated ration mainly due to apparent nutritional sufficiency, higher digestibility, and ready acceptance by the larval stage of fish and shellfish species [27]. Several studies have revealed the application of fairy shrimps and their premium nutritional value as potential and new food sources for the ever-developing aquaculture industry [27,28,29]. Fairy shrimps might be valuable alternatives for the widely used brine shrimp Artemia; however, other than very few reports, relatively little is known regarding their life-cycle characteristics [6,30]. Therefore, enhancing the growth performance and other physiological features of such species under predefined environmental conditions, such as photoperiod, is important to improve the live prey production and aquacultural application.

The present study was aimed at investigating the effects of photoperiod manipulation on the hatching rate, growth performance, activity of digestive enzymes, antioxidative status, proximate body composition, and amino-acid and fatty-acid profiles of fairy shrimp (*B. orientalis*). The results can serve to enlighten the physiology of the species under various photoperiods, with applications in the development of mass production protocols for the species.

## 2. Materials and Methods

### 2.1. Hatching Experiment

Dehydrated cysts of *B. orientalis* were hatched using tap water (EC: 265 μS cm^−1^, pH: 8.2) in different photoperiods (24:0, 16:08, 12:12, and 0:24 light/dark cycles) under 2000 lux fluorescent light and an optimum temperature of 21 °C [9]. Hatching under experimental photoperiods was performed in triplicate using 50 dormant eggs per each trial. The eggs were incubated in multi-well (30 mL) plastic trays for a period of 10 days. During this period, the emerged nauplii were counted and removed from the tray on a daily basis. At the end of the hatching trial, dormant unhatched eggs were tested for their viability by testing for the presence of a yolky embryo according to the method used by Atashbar et al. [9].

### 2.2. Breeding Conditions

Two hundred newly hatched nauplii were gently transferred to each 1.5 L cylindroconical glass container (as an experimental unit) filled with 1 L of well-aerated tap water and reared for 17 days under four different photoperiods (24:0, 16:08, 12:12, and 0:24 light/dark) in triplicate. The medium was renewed on days 8, 11, 14, and 17 of the breeding period. The experimental units were mildly aerated using a fine bubbler, and fairy shrimps were fed *Haematococcus* sp. (Table 1, see Excel file in Supplementary Materials for all tables) daily at a concentration of 18 × 10^6^ cells/mL [31]. The algae were cultured in 4 L cylindrical containers using 3NBBM as the culture medium (pH: 8, temperature: 21 ± 1 °C, and 3500 lux).

### 2.3. Survival and Growth

Survival and total length were determined on days 8, 11, 14, and 17. To determine survival rate for each experimental unit, all individuals were collected using a 200 µm net and counted. For determining growth, the total body length of 12 individuals from each replicate were measured from the most anterior part of the head to the last abdominal segment (telson) using a light microscope equipped with a phototube and micrometer. Drawings were later digitized using a digitizer (Summa Sketch TM III, Vancouver, BC, Canada) connected to a computer.

### 2.4. Digestive Enzyme Activity

#### 2.4.1. Sample Preparation

To assay digestive enzymes (alkaline protease, alpha-amylase, and lipase) activity, three pooled samples of 0.5 g wet weight were randomly taken from each treatment. The samples were homogenized )5:1 *w*/*w*) in 50 mM Tris-HCL buffer, pH 7, using a homogenizer for 1.5 min on ice. Lastly, the homogenates were centrifuged for 20 min at 4 °C with a speed of 1000× *g*. The supernatant was stored at −80 °C to assay soluble protein content and enzyme activity [32].

#### 2.4.2. Amylase Activity

The amylase activity was determined using Bernfeld’s [33] method. In brief, 50 µL of crude enzyme extract was incubated with a 3 g/L starch solution (in 66 mM Na_2_PO_4_) for 20 min at 25 °C. The enzyme reaction was halted using 20 µL of 1 N HCl. Lastly, the resultant reduced sugar was quantified using 3,5-dinitrosalicylic acid, as a chromogenic agent, at 580 nm. One unit of α-amylase activity was defined as mg of starch hydrolyzed per minute.

#### 2.4.3. Lipase Activity

The lipase activity of the supernatants was spectrophotometrically determined by *p*-nitrophenyl myristate hydrolysis [34]. Every assay (0.5 mL) included 0.53 mM nnitrophenyl myristate, 0.25 mM 2-methoxyethanol, 5 mM sodium cholate, and 0.25 M Tris-HCl (pH 9.0). The mixture incubated with 50 µL of crude enzyme extract for 15 min at 25 °C. The hydrolytic reaction was stopped via addition of 0.7 mL of acetone/*n*-heptane (5:2, *v*/*v*) solution. Afterward, the cocktail was vigorously mixed and centrifuged at 6080× *g* for 2 min. The absorbance of the lower aqueous layer was recorded at 405 nm. Unit enzyme activity was calculated as 1 μmol of *n*-nitrophenol per minute.

#### 2.4.4. Alkaline Protease Activity

Alkaline protease activity was measured using azocasein substrate solution (2%, pH = 7.5) in 50 mM Tris-HCl [35]. First, 20 µL of the supernatant was incubated with 0.5 mL of the substrate solution at 25 °C for 10 min. After incubation, 0.5 mL of trichloroacetic acid (TCA) solution was added to terminate the reaction. The resultant mixture was centrifuged at 6500× *g* for 5 min. Lastly, the resultant supernatant was transferred to a microplate, and the absorbance was read by a spectrophotometer at 440 nm. The specific activity of alkaline protease was calculated according to the incubation time (10 min) and the soluble protein content of the supernatant (mg) [35].

### 2.5. Soluble Protein Quantification

The crude extract soluble protein content was measured using Bradford’s [36] method. Serum albumin (BSA) was used as the standard. In brief, 5 µL of crude extract and 250 µL of Bradford reagent were mixed and incubated for 7 min at 25 °C. Lastly, the optical density of the mixture was spectrophotometrically recorded at 595 nm.

### 2.6. Antioxidant Status

The following enzymatic activities were measured in fairy shrimp homogenates: superoxide dismutase (SOD, converts O_2_^−^ to H_2_O_2_), catalase (CAT, reduces H_2_O_2_ to water), and glutathione peroxidase (GPx, detoxifies H_2_O_2_ or organic hydroperoxides produced, e.g., by lipid peroxidation). All enzymatic assays were measured using 2 µL of crude enzyme extract [37]. The final results for enzymatic activities were normalized by protein content.

### 2.7. Proximate Composition

Proximate analysis was carried out according to the methodology of the Association of Official Analytical Chemists [38]. Crude protein was determined by the Kjeldahl method using an auto Kjeldahl system. Crude lipid was determined by ether extraction using a Soxhlet extractor. Ash content was determined using a muffle furnace at 550 °C for 5 h.

### 2.8. Fatty-Acid Profile

The fatty-acid composition was analyzed by producing methyl esters according to Folch et al. [39], Ichihara et al. [40], and Lepage and Roy [41] with some modification. In brief, 0.5 g of fresh biomass was homogenized in chloroform/methanol (2:1, *v*/*v*) for lipid extraction. Methyl esters were prepared by transmethylation using 2 M KOH in methanol and *n*-hexane. The fatty-acid composition was analyzed by a gas chromatograph (GC, Agilent technologies 7890 N, Wilmington, NC, USA) equipped with a flame ionization detector and a cyanopropyl-phenyl capillary column. Lastly, identification of the fatty acids was performed by comparing their retention time with those of an external commercial standard mixture (GLC-68d, Nu-Chek Prep., Elysian, MN, USA).

### 2.9. Amino-Acid Analysis

The amino-acid profiling of samples was carried out by treating 200 mg of freeze-dried fresh biomass with 10% trichloroacetic acid (TCA) to completely precipitate all protein contents. The precipitates were consecutively rinsed using 7% TCA, ethanol, chloroform–methanol (3:1), and diethyl ether solutions. Then, the precipitate was collected by centrifugation. Lastly, the precipitates were hydrolyzed using 6 N hydrochloric acid at 110 °C for 5 h. The hydrolysates were freeze-dried and derivatized using O-phthaldialdehyde reagent [42]. Amino-acid composition was determined at 338 nm using an HPLC system (Agilent 1100 Series, Waldbronn, Baden-Württemberg, Germany) equipped with a C18 column (4.6 × 150). The solvents were introduced at a flow rate of 0.50 mL/min at 40 °C (sodium acetate buffer and acetonitrile buffer adjusted to a pH 7.2).

### 2.10. Data Analysis

Statistical analysis was conducted with SPSS 23. One-way analysis of variance (one-way ANOVA) followed by Tukey’s HSD test was used to identify significant variations. Between-group differences were statistically considered significant at the 0.05 level. Data were expressed as the mean ± SD.

## 3. Results

### 3.1. Hatching Rate

**Figure 1:**
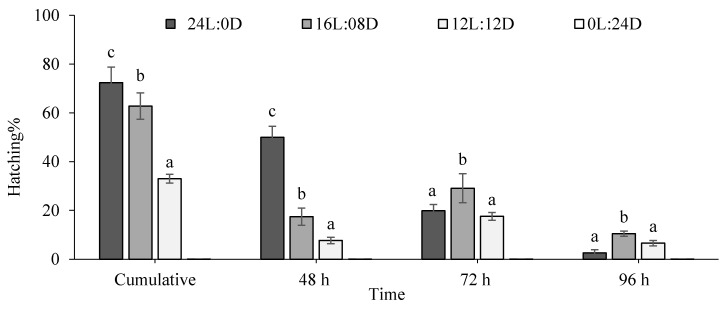
Comparison of hatching characteristics of *B. orientalis* under different photoperiod regimes (mean ± SD, *n* = 3). Different superscript letters denote statistically significant differences at each timepoint (*p* < 0.05).

The hatching rates of fairy shrimp under different photoperiods are presented in Figure 1. The hatching rates showed significant differences under different photoperiods and days of incubation (*p* < 0.05). The *B. orientalis* cumulative hatching rate was highest at 24L:0D (72.41% ± 6.35%) and the lowest at 12L:12D (32.97% ± 2.59%). No hatching occurred under the 0L:24D regime. According to the results, the initial hatching rate (48 h) was the highest at 24L:0D (50.00% ± 4.50%) in comparison to other experimental groups and the lowest at 12L:12D (7.69% ± 1.30%), while the highest hatching rate of cysts at 12L:12D and 16L:08D was observed after 72 h of incubation.

### 3.2. Growth and Survival

The effects of different photoperiods on the growth and survival rate of *B. orientalis* are presented in Table 2 at the end of a 17 day trial. There were no significant differences amongst various experimental groups in terms of total body length on days 8, 11, and 15 (*p* > 0.05). However, individuals reared under a 0L:24D regime (13.31 ± 0.78 mm) showed significantly higher total body length on day 17 (*p* < 0.05). Results also showed that there were no considerable differences regarding daily growth rate from days 1 to 8, while those individuals stocked at 0L:24D showed a significantly higher daily growth rate from day 8 onward (1.03 mm/day) (*p* < 0.05, Table 1). No significant differences were detected in terms of the specific growth rate (SGR) and average final body weight of animals grown under different photoperiods (*p* > 0.05).

Sexual maturity rate was significantly higher at 24L:0D (25.75% ± 1.23%) in comparison to other experimental groups, and the lowest rate was recorded at 0L:24D (11.81% ± 1.19%) (*p* < 0.05).

**Figure 2:**
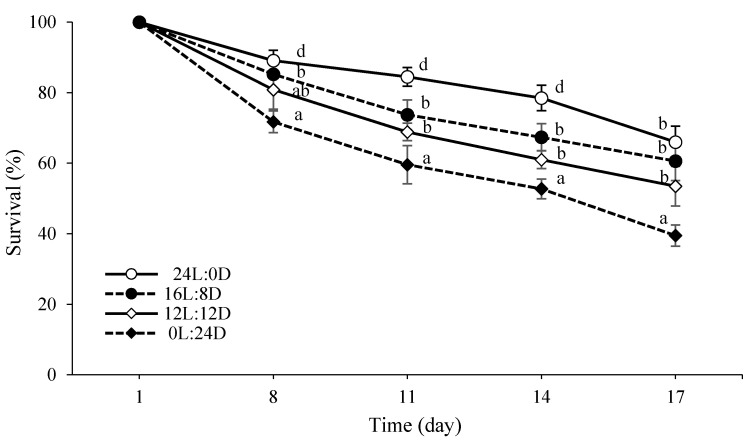
Comparison of survival rate of *B. orientalis* under different photoperiod regimes (mean ± SD, *n* = 3). Different superscript letters denote statistically significant differences at each timepoint (*p* < 0.05).

The results indicated that the survival rate of the fairy shrimp significantly decreased with increasing length of the dark period (*p* < 0.05, Figure 2). A significantly higher survival rate was observed in the group reared at 24L:0D (*p* < 0.05, Figure 2). The lowest survival rate was recorded under the 0L:24D regime (*p* < 0.05).

### 3.3. Digestive Enzyme Activity

**Figure 3:**
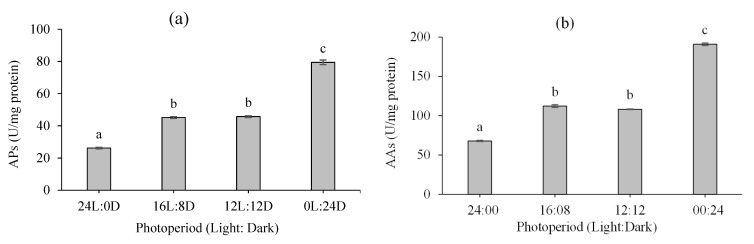

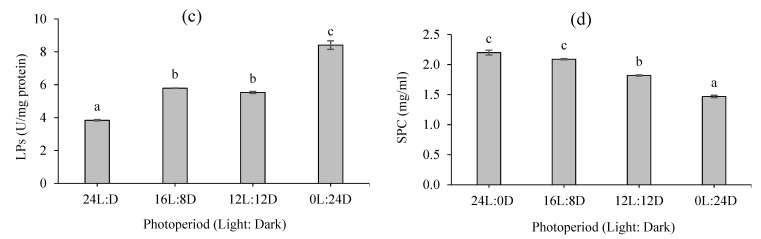
Effect of different photoperiods on digestive enzyme activity of *B. orientalis* at the end of the experimental period (mean ± SD, *n* = 3): (**a**) alkaline protease (AP), (**b**) alpha-amylase (AA), (**c**) lipase (LP), and (**d**) soluble protein content (SPC). Different superscript letters denote statistically significant differences at *p* < 0.05.

The digestive enzyme activity of fairy shrimp under various photoperiods are presented in Figure 3a–d. Overall, the highest and lowest digestive enzyme activities, including alkaline protease (Figure 3a), alpha-amylase (Figure 3b), and lipase (Figure 3c), were observed at 0L:24D and 24L:0D, respectively (*p* < 0.05). However, there were no significant differences between the 16L:08D and 12L:12D photoperiods regarding digestive enzyme activity (*p* > 0.05). In addition, the highest and lowest soluble protein content (Figure 3d) was observed at 24L:0D (2.10 ± 0.01) and 0L:24D (1.44 ± 0.02), respectively (*p* < 0.05).

### 3.4. Antioxidant Status

**Figure 4:**
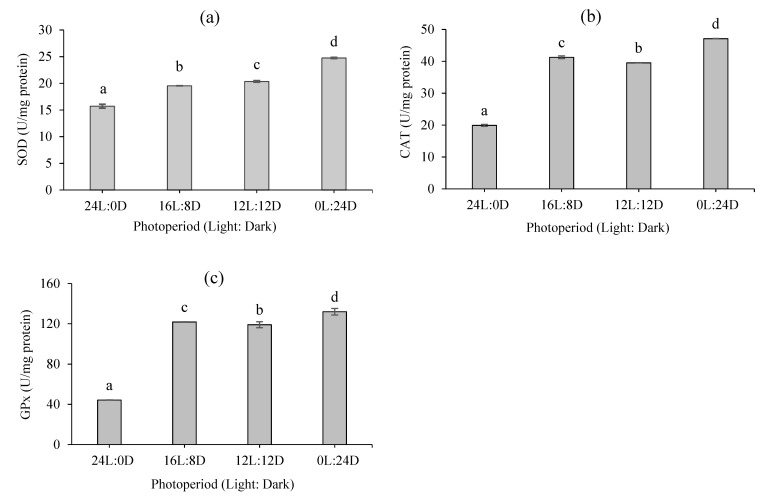
Antioxidative enzyme activity of *B. orientalis* at the end of the trial (mean ± SD, *n* = 3): (**a**) superoxide dismutase (SOD), (**b**) catalase (CAT), and (**c**) glutathione peroxidase (GPx). Different superscript letters denote statistically significant differences at *p* < 0.05.

The effects of different photoperiods on the antioxidant status of fairy shrimp are depicted in Figure 4a–c. The highest and lowest antioxidant enzyme activities, including superoxide dismutase (SOD, Figure 4a), catalase (CAT, Figure 4b), and glutathione peroxidase (GPx, Figure 4c), were observed at 0L:24D and 24L:0D, respectively (*p* < 0.05). However, no significant differences were detected between the 16L:08D and 12L:12D photoperiods regarding such enzyme activity (*p* > 0.05).

### 3.5. Proximate Body Composition

The proximate body compositions of *B. orientalis* grown under different photoperiods are presented in Table 3. The crude protein (56.15 ± 1.23) and lipid (14.51 ± 0.54) contents of individuals reared at 24L:0D were significantly higher (*p* < 0.05, Table 2). The significantly lowest protein and lipid contents were observed at 0L:24D (50.33 ± 0.64) and 12L:12D (5.05 ± 0.40), respectively (*p* < 0.05, Table 3). However, the *ash content* of various experimental groups did not significantly differ (*p* > 0.05, Table 3).

### 3.6. Fatty-Acid Profile

The fatty-acid profile of fairy shrimp grown under various photoperiods is presented in Table 4. The highest and lowest contents of saturated fatty acids (SFAs) and docosahexaenoic acid (DHA) were observed at 12L:12D and 16L:8D, respectively (*p* < 0.05, Table 4). Furthermore, the highest and lowest contents of polyunsaturated fatty acids (PUFAs) and α-linolenic acid (ALA) were observed at 16L:8D and 24L:0D, respectively (*p* < 0.05). In contrast, the contents of monounsaturated fatty acids (MUFAs), linoleic acid (LA), and eicosapentaenoic acid (EPA) at 24L:0D were significantly higher in comparison to other experimental groups (*p* < 0.05).

### 3.7. Amino-Acid Analysis

As presented in Table 5, there were differences among various experimental groups in terms of total amino acids (TAAs) and essential amino acid contents (EAAs). Extension of the lighting period, especially in the 16L:8D group, resulted in a noticeable increase in the body contents of all amino acids compared to other treatments. Table 6 compares the essential amino-acid profile (% dry weight) of *B. orientalis* and common live preys used in ornamental freshwater fish nutrition with reference to dietary essential amino-acid requirements of common freshwater fish species. It is evident that *B. orientalis* possesses all essential amino acids required by freshwater fish species. In comparison to Artemia, bloodworm, and Moina, the three most common live food organisms, the lysine content of *B. orientalis* was higher, especially in animals kept under 16L:8D culture conditions. Furthermore, its essential amino-acid content was considerably higher than bloodworm, which is a very popular organism in freshwater ornamental fish production.

## 4. Discussion

The diapause stage of many arthropods has been known to resume active development when exposed to one or more environmental stimuli, including light. Our results showed that hatching was more successful with an increased lighting cycle, with any decreases in light exposure negatively affecting various hatching indices, including cumulative hatching, the initial hatching fraction, and time of the maximum hatching of the fairy shrimp. The highest cumulative hatching rate was recorded at 24L:0D. Contrary to our results, relatively lower hatching percentages and efficiencies were obtained in the *A. urmiana* cysts in complete darkness [13]. Wang et al. [24] showed that a shorter photoperiod (6L:18D) could induce diapause in the parthenogenetic Artemia. Light-induced hatching has been widely reported in branchiopod crustaceans, with different light conditions (in terms of light intensity and photoperiod) resulting in different hatching success [21,22]. It is well known that the hatching percentage, efficiency, and rates of crustacean cysts vary from one species to another, and variations might even be expected among different batches of the same strain [21]. 

It has been demonstrated that the growth performance of various aquatic animals can be enhanced by continuous lighting or extending the light cycle, for example, striped knifejaw, *Oplegnathus fasciatus* [12], *Artemia urmiana* [13], Mudcrabs, *Scylla serrata* [14], barfin flounder larvae, *Verasper moseri* [45], longarm *river prawn*, *Macrobrachium tenellum* [46], and spotted sea bass, *Lateolabrax maculatus* [15]. In the present study, manipulating the photoperiod (especially 16L:8D) imperceptibly increased the growth performance (body weight and specific growth rate) of the fairy shrimp. Our results are in a good agreement with Bermudes and Ritar [47], who reported a slight increase in molt increment (mm) in spiny lobster, *Jasus edwardsii*, larvae (stage III–IV) reared under a 6–18 h lighting period. In contrast, some species, including decapod larvae, *Carcinus maenas* [48], and *Thenus orientalis* [49], showed a consistent growth response to photoperiod throughout larval development. This study also revealed that extending the photoperiod from 0L to 24L remarkably improved the survival rate of the fairy shrimp to the extent that the highest survival was observed at 24L:0D. Contrary to our results, Santos-Romero et al. [46] showed that photoperiod had no significant effect on the survival of *Macrobrachium tenellum* larvae. Increased growth rate could be attributable to the fact that aquatic animals might be more active under such conditions and show increased foraging activity [47,50]. In addition, it has been shown that nonselective filter feeder organisms such as brine shrimp might take up any suspended particles when appropriate feed items are absent or scarce [51], implying that any seasonal changes in feed/prey availability might also affect zooplankton growth. Furthermore, rhythmic environmental factors can regulate internal physiological processes. For instance, it has been suggested that photoperiod affects the ecdysial rhythm in crustaceans. These are possible explanations for the elevated rate of development and growth observed at different stages of larval crustaceans reared under different photoperiods [41,47]. Meanwhile, we observed that the fairy shrimps grew significantly faster at 0L:24D from day 8 onward, as total length was increased compared to other experimental groups. Our results are in a good accordance with Pormehr Yabandeh et al. [10], who witnessed a *significant increase* in *growth rates* with decreasing density for the same species in the culture unit. Similarly, we observed that, concomitant with decreasing lighting period, the survival rate was decreased, i.e., the fairy shrimp density was decreased. Density-dependent limiting factors such as competition for resources (e.g. food and space) probably increased with a higher population density, eventually limiting the growth rate of individuals.

Proximate body composition analyses of *B. orientalis* showed that body crude protein content was higher under the 24L:0D regime compared to the other treatments. Protein contents obtained in this study (ranging from 50.33% to 56.15%) were similar to the values (49.7–52.0%) reported in *B. orientalis* [10] and *Streptocephalus siamensis* (50.2%) [52] under different feeding and laboratory conditions. In the present study, the body lipid content of *B. orientalis* was significantly higher at 24L:0D. The lipid content of the animals ranged from 5.05% to 14.51%, which differs slightly from the previous reports on *B. orientalis* (9.4–10.0%) [10] and three Thai fairy shrimp species, *S. siamensis, B. thailandesis* [53], and *S. sirindhornae* (6.1%, 7.6%, and 9.3%, respectively) [52]. Such different results might be due to various feeding and laboratory conditions.

Digestive enzyme activity has been used as an important indicator for evaluating the digestive capacity and nutritional status of different animals including crustaceans [54,55]. In addition, an appreciation of the digestion process is vital in terms of understanding the feeding and nutritional ecology of animals [53]. The effects of light/dark cycle on the digestive enzyme activity of crustaceans, especially planktonic species including brine or fairy shrimps, have not been studied in detail. For instance, the effect of temperature and photoperiod on the digestive enzyme activity of juvenile *M. tenellum* was investigated [46]. It was shown that, among different enzymes (trypsin, chymotrypsin, lipases, amylases, and chitinase), only chitinase proved to be significantly different in shrimps stocked under the 14L:10D regime. Our study revealed that the 0L:24D photoperiod significantly increased the activity of all three enzymes (alkaline protease, alpha-amylase, and lipase). One can assume that such an increase in digestive enzyme activity might be attributable to the higher feed intake in the 0L:24D regime. In the present study, the culture volume and quantity of the daily algae provision were constant for all experimental units such that the group kept under a 0L:24D lighting cycle could benefit from a higher quantity of feed due to the increased mortality rate under such a cycle. Such an inference was evident regarding the daily growth rate from day 8 onward among various experimental groups. Regarding the effect of feed intake on digestive enzyme activity, a close relationship was reported between proteolytic enzyme activity and feeding rates in actively diapausing cyclopoids and active cyclopoids of similar size [56]. In this sense, it has been thoroughly discussed that larval crustaceans might rely on vision for predator avoidance, migration, and selection of appropriate settlement habitats; however, there is no consensus on the role of vision in feeding [57]. Nevertheless, the exact mechanism underlining this process is far from being understood and requires further investigation.

Antioxidant enzymes, mainly including superoxide dismutase (SOD), catalase, and glutathione peroxidase (GPx), are coordinately involved in neutralizing reactive oxygen species (ROS). ROSs are constantly formed during normal cellular metabolism [58]. However, ROS overproduction can result in biomolecule damage, mostly lipids, proteins, and DNA [59]. It has been shown that light intensity could influence the antioxidant system in aquatic animals (mainly fish), including blunt snout bream (*Megalobrama amblycephala*) [60], Senegalese sole (*Solea senegalensis*) [61], orange-spotted grouper (*Epinephelus coioides*) [62], and goldfish (*Carassius auratus*) [63]. Our results revealed that the oxidative stress of fairy shrimp significantly increased in complete darkness (0L:24D), in line with findings on Gibel carp (*Carassius auratus*), in which the antioxidant capacity and the SOD, GP, and metabolite glutathione contents were highest in the short-day groups (0L:24D, 4L:20D, and 8L:16D) [64]. Nevertheless, in half-smooth tongue sole (*Cynoglossus semilaevis*), the enzyme activity of SOD was higher at 24L:0D than that at 0L:24D, 6L:18D, and 18L:6D [64,65]. In addition, it is plausible that increased daily feed provision per individual fairy shrimp under the 0L:24D regime could have subsequently resulted in increased heat increment of feeding mainly associated with nutrient digestion and uptake [66]. Consequently, the higher metabolic rate might have led to higher oxygen uptake and, therefore, an increase in the production rate of ROS in the body. 

Fatty acids are involved in organismal adaptive responses to any changes in the environment, participate in cellular energy provision and physiological processes, and regulate biochemical reactions [67]. It has long been recognized that culture conditions play a major role in determining the quantity and quality of the fatty-acid profile in aquatic animals [64,67,68]. Our results showed that the fatty-acid content of fairy shrimp was affected by the light/dark cycle. There is no information regarding the possible effect of photoperiod on the fatty-acid profile of intertidal invertebrates; however, it has been reported that any increase in lighting period might affect the fatty-acid reserves of suspension feeders in such a way that PUFA reserves decrease most likely due to metabolic changes associated with reproductive development [69]. Such a decrease in carcass PUFA contents, especially under the 24L:0D regime, was evident. At the same time, the Eicosapentaenoic acid (EPA) and Docosahexaenoic acid (DHA) contents of fairy shrimps grown under 24L:0D and 12L:12D regimes increased, implying some physiological preparations for important life-cycle events such as reproduction. However, the reproductive development of the fairy shrimp under such conditions warrants detailed studies in the future, as evidently reflected by the sexual maturity on day 17 (Table 2) in groups grown under continuous light. From an aquaculture point of view, the higher EPA and DHA contents of fairy shrimp under 24L:0D and 12L:12D regimes could indicate their nutritional quality in meeting essential fatty-acid requirements of fish larvae [70].

Our results revealed variations in the total percentages of essential amino-acid composition among the animals cultured under different photoperiods. EAAs provided more than 51% of the total amino-acid content of *B. orientalis*. It has been previously shown that factors other than diet, including animal source and genetic moiety of the animal, can affect amino-acid profile [70]. Similarly, our results revealed that photoperiod was able to affect the amino-acid profile of *B. orientalis* to the extent that rearing under a 16L:8D regime resulted in a noticeable increase in EAA content of the animal. In addition, the EAA content of *B. orientalis* at 16L:8D could meet the general freshwater fish requirements. Similar to our findings, a higher total free amino-acid concentration was reported in *Orconectes virilis* and *Gammarus pseudolimnaeus* in May in comparison to other months, when the natural light period is closer to the 16L:8D treatment [71,72]. We also noticed that continuous lighting resulted in lower contents of total TAAs and nonessential amino acids such as glutamic acid, aspartic acid, and serine in *B. orientalis*. Similarly, the amount of serine in narrow-clawed crayfish (*Pontastacus leptodactylus*, Eschscholtz, 1823) was significantly reduced under continuous lighting [73]. A higher body essential amino-acid content (*ca.* >51%) could be indicative of the suitability of *Haematococcus* sp. as feed for *B. orientalis*, as previously reported in the feeding of *Streptocephalus sirindhornae* with various diets including *Chlorella vulgaris*, *Saccharomyces cerevisiae*, and *Rhodopseudomonas faecalis* [74]. However, their proportions varied among different treatments mainly due to changes in the protein metabolism or energetic status of the organism [72].

## 5. Conclusions

The current research provided updated life history features with important implications for fairy shrimp species, particularly *B. orientalis*. In general, our results revealed that an extended light period provides better environmental conditions for hatching, growth, and survival of the species. Those individuals kept under constant lighting contained more than twofold lipid content compared to that detected in the 16L:8D and 12L:12D groups. Meanwhile, the 16L:8D light/dark cycle might be an optimal condition in terms of the growth performance and physio-biochemical properties of *B. orientalis*. However, for higher hatching efficiency and better hatching synchrony, constant lighting (i.e., 24L:0D) is recommended. In addition, this information might be important regarding the possible use of such zooplankton in the early life stage of freshwater fish nutrition. Since prey size is an important factor in terms of feed intake and energy expenditure of predator, it could be possible to provide suitable prey size for different life stages and species of freshwater ornamental fish by varying the harvesting time of *B. orientalis* in a 17 day cycle to gain better growth performance and survival rate. This size flexibility is not manageable in small live food organisms, including *Daphnia* and rotifer. In comparison to different live feed items including Artemia, fairy shrimp possessed a favorable amino-acid profile with respect to the amino-acid requirements of various freshwater fish species. Whereas Artemia is widely used as live feed for ornamental fish culture, the zooplankton cannot survive in a freshwater environment for a long time. Furthermore, they would also quickly sink to the bottom of the rearing tank and become inaccessible to pelagic fish larvae unless constantly supplied. In conclusion, regarding these different characteristics, fairy shrimp is a promising live prey for the culture of various freshwater aquatics.

## Figures and Tables

**Table 1:** Fatty-acid profile and proximate composition of *Haematococcus* sp. used to feed *B. orientalis*.

Fatty Acids Profile (% Area of Total Fatty Acids)	
C 14	3.40 ± 1.14
C 14:1n-5	1.84 ± 0.18
C 16	2.94 ± 0.04
C 16:1n-7	1.56 ± 0.18
C 18	14.87 ± 1.15
C 18:1n-9	2.37 ± 0.24
C 18:1n-7	-
C 18:2n-6	4.03 ± 0.04
C 18:3n-3	4.55 ± 0.05
C 20	0.55 ± 0.12
C 20:4n-6	0.48 ± 0.27
C 20:3n-3	-
C 20:5n-3	0.69 ± 0.02
C 22	2.91 ± 0.16
C 24	0.28 ± 0.01
C 24:1n-9	0.47 ± 0.02
SFA	24.95 ± 2.62
MUFA	6.24 ± 0.62
PUFA	9.74 ± 0.38
Proximate composition (% dry matter)	
Crude protein	13.53 ± 1.37
Crude lipid	43.58 ± 1.77
Ash	7.18 ± 2.01

**Table 2:** Total length (TL), daily growth, specific growth rate (SGR), and final body weight of *B. orientalis* under different photoperiods (mean ± SD, *n* = 3).

Indices	Photoperiod (Light/Dark)			
	24L:0D	16L:08D	12L:12D	0L:24D
TL on day 1	0.58 ± 0.04 ^a^	0.58 ± 0.04 ^a^	0.58 ± 0.04 ^a^	0.58 ± 0.04 ^a^
TL on day 8	4.03 ± 0.20 ^a^	3.86 ± 0.31 ^a^	3.59 ± 0.17 ^a^	4.00 ± 0.11 ^a^
TL on day 11	7.72 ± 0.23 ^a^	6.81 ± 0.48 ^a^	5.82 ± 0.11 ^a^	6.41 ± 0.28 ^a^
TL on day 15	11.32 ± 0.50 ^a^	10.37 ± 0.31 ^a^	11.31 ± 0.82 ^a^	11.19 ± 0.81 ^a^
TL on day 17	11.62 ± 0.57 ^a^	11.70 ± 0.31 ^ab^	12.73 ± 0.54 ^ab^	13.31 ± 0.78 ^b^
Daily growth rate from day 1–8 (mm/day) *	0.43 ± 0.02 ^a^	0.41 ± 0.03 ^a^	0.38 ± 0.02 ^a^	0.43 ± 0.01 ^a^
Daily growth rate from day 9–17 (mm/day)	0.84 ± 0.04 ^a^	0.87 ± 0.00 ^ab^	1.02 ± 0.04 ^b^	1.03 ± 0.07 ^b^
SGR (% body weight/day) **	16.52 ± 1.01 ^a^	17.18 ± 0.86 ^a^	16.63 ± 0.32 ^a^	16.81 ± 0.85 ^a^
Average body weight (mg)	17.09 ± 2.28 ^a^	18.65 ± 1.06 ^a^	17.51 ± 2.87 ^a^	18.14 ± 2.64 ^a^
Sexual maturity on day 17 (%) ***	25.75 ± 1.23 ^c^	17.59 ± 0.78 ^bc^	18.06 ± 2.33 ^bc^	11.81 ± 1.19 ^a^

Different superscript letters within each row denote statistically significant differences at *p* < 0.05. * Daily growth rate = (length on day 8 − length on day 1)/8; ** SGR% = ((Ln(W2) − Ln(W1))/17) × 100; *** sexual maturity on day 17 = (number of adults/total number) ×100.

**Table 3:** Proximate body composition (% dry matter) of *B. orientalis* cultured under different photoperiods (mean ± SD, *n* = 3).

Proximate Composition (% Dry Matter)		Photoperiod (Light/Dark)		
	24L:0D	16L: 8D	12L:12D	0L:24D
Crude protein	56.15 ± 1.23 ^b^	53.87 ± 1.70 ^b^	53.46 ± 2.62 ^b^	50.33 ± 0.64 ^a^
Crude lipid	14.51 ± 0.54 ^c^	6.91 ± 0.66 ^a^	5.05 ± 0.40 ^a^	12.61 ± 0.86 ^b^
Ash	12.73 ± 1.40 ^a^	11.32 ± 1.46 ^a^	12.86 ± 1.14 ^a^	11.69 ± 0.87 ^a^

Different superscript letters within each row indicate statistically significant differences at *p* < 0.05.

**Table 4:** Effect of different photoperiods on fatty-acid profile of *B. orientalis* at the end of the trial (mean ± SD, *n* = 3).

Fatty Acids Profile	Photoperiod (Light: Dark)			
(% area of total fatty acids)	24L:0D	16L: 8D	12L:12D	0L:24D
SFA	24.87 ± 0.23 ^b^	21.40 ± 0.40 ^a^	27.84 ± 0.16 ^d^	24.18 ± 0.19 ^c^
MUFA	28.28 ± 0.27 ^d^	16.61 ± 0.10 ^a^	25.06 ± 0.07 ^c^	18.50 ± 0.08 ^b^
PUFA	46.21 ± 0.22 ^a^	54.86 ± 0.06 ^d^	46.81 ± 0.06 ^b^	52.43 ± 0.13 ^c^
PUFA *n*-3	13.91 ± 0.11 ^a^	18.68 ± 0.33 ^d^	16.48 ± 0.22 ^c^	15.22 ± 0.23 ^b^
PUFA *n*-6	32.30 ± 0.11 ^b^	36.19 ± 0.40 ^c^	30.33 ± 0.28 ^a^	37.21 ± 0.10 ^d^
*n*-3/*n*-6	0.43 ± 0.00 ^a^	0.52 ± 0.01 ^b^	0.54 ± 0.01 ^b^	0.41 ± 0.01 ^a^
EPA	1.25 ± 0.02 ^d^	0.72 ± 0.01 ^b^	1.18 ± 0.03 ^c^	0.34 ± 0.01 ^a^
DHA	2.29 ± 0.01 ^c^	0.54 ± 0.00 ^a^	2.30 ± 0.02 ^c^	1.61 ± 0.02 ^b^
EPA/DHA	1.84 ± 0.03 ^b^	0.75 ±0.01 ^a^	1.95 ± 0.06 ^b^	4.71 ± 0.17 ^c^

Different superscript letters within each row indicate statistically significant differences at *p* < 0.05. Saturated fatty acid (SFA), Monounsaturated fatty acids (MUFAs), Polyunsaturated fatty acids (PUFAs), Eicosapentaenoic acid (EPA) and Docosahexaenoic acid (DHA).

**Table 5:** Amino-acid composition of *B. orientalis* (mg/g dry weight) at the end of the trial (mean ± SD, *n* = 3).

Amino Acid	Photoperiod (Light/Dark)			
	24L:0D	16L:8D	12L:12D	0L:24D
Alanine	33.78	40.10	40.60	36.50
Arginine	31.86	43.60	38.30	37.00
Aspartic acid	49.50	61.70	59.50	50.90
Glutamic acid	70.79	89.70	85.10	77.50
Glycine	26.79	32.30	32.20	30.70
Histidine	12.40	16.50	14.90	13.10
Isoleucine	22.05	27.30	26.50	24.10
Leucine	38.18	47.70	45.90	42.70
Lysine	43.84	55.20	52.70	45.60
Methionine	9.82	12.30	11.80	10.30
Phenylalanine	24.62	29.30	29.60	28.30
Serine	23.96	30.60	28.80	25.20
Threonine	26.04	31.20	31.30	30.80
Tryptophan	6.24	7.50	7.50	2.30
Tyrosine	21.30	26.90	25.60	14.30
Valine	26.87	32.00	32.30	29.80
TAAs	468.04	583.9	562.6	499.1
TEAAs *	241.92	302.6	290.8	264

* Total amino acids (TAAs); Total essential amino acids (TEAAs). Similar to other animals, arginine, histidine, isoleucine, leucine, lysine, methionine, phenylalanine, threonine, tryptophan, and valine were considered as essential amino acids.

**Table 6:** Essential amino-acid profile (% dry weight) of *B. orientalis*, reared under different light/dark cycles, and common live preys used in ornamental freshwater fish nutrition, along with dietary amino-acid requirements of common freshwater finfish. Amino acid (AA), Total amino acid (TAA) and Total essential amino acid (TEAA).

	*B. orientalis* Reared under Different Photoperiods													
AA	24l:0D	16L:8D	12L:12D	0L:24D	Artemia *	Blood worm *	Moina *	Rainbow trout **	Nile tilapia **	Common carp **	Catla (Fry) **	Marigal **	Channel catfish **	Goldfish larvae ***
Arginine	3.19	4.36	3.83	3.70	6.2	2.81	5.1	1.8	1.2	1.7	1.92	1.8	1.0	0.30
Lysine	4.38	5.52	5.27	4.56	4.0	3.05	4.31	1.9	1.4	2.2	2.49	2.3	1.2	0.36
Histidine	1.24	1.65	1.49	1.31	1.6	1.41	1.57	0.6	1.0	1.0	0.98	0.9	0.4	0.12
Isoleucine	2.21	2.73	2.65	2.41	2.4	2.5	2.55	1.4	1.8	1.0	0.94	1.7	0.6	0.18
Leucine	3.82	4.77	4.59	4.27	5.0	4.22	5.1	3.4	1.9	1.3	1.48	1.5	0.8	0.24
Valine	2.69	3.20	3.23	2.98	3.2	3.28	3.73	1.6	1.6	1.4	1.42	1.5	0.7	0.21
Methionine	0.98	1.23	1.18	1.03	1.6	2.19	1.96	0.8	0.8	0.8	1.42	1.2	0.6	0.18
Phenylalanine	2.46	2.93	2.96	2.83	3.4	3.83	3.53	0.7	1.1	1.5	1.48	1.3	0.5	0.15
Threonine	2.60	3.12	3.13	3.08	3.4	2.58	3.33	1.1	1.1	1.5	1.98	1.8	0.5	0.15
Tryptophan	0.62	0.75	0.75	0.23	-	-	-	0.2	0.28	0.3	0.38	0.4	0.1	-
*TEAA*	*24.19*	*30.26*	*29.08*	*26.40*	*30.8*	*25.87*	*31.18*	-	-	-	-	-	-	-
*TEAA/TAA*	*51.69*	*51.82*	*51.69*	*52.90*	*51.00*	*54.84*	*49.58*	-	-	-	-	-	-	-

* [42], ** [43], *** [44].

## Data Availability

Not applicable.

## Supplementary Materials

The following are available online at https://www.mdpi.com/article/10.3390/biology10080695/s1.

## References

[1] Eng L.L., Belk D., Eriksen C.H. (1990). Californian Anostraca: Distribution, habitat and status. J. Crustac. Biol..

[2] Eriksen C., Belk D. (1999). Fairy Shrimps of California’s Puddles, Pools, and Playas.

[3] Manca M., Mura G. (1997). On *Branchinecta orientalis* Sars, 1901 (Anostraca) in the Himalayas. Hydrobiologia.

[4] Horváth Z., Vad C.F., Vörös L., Boros E. (2013). Distribution and conservation status of fairy shrimps (Crustacea: Anostraca) in the astatic soda pans of the Carpathian basin: The role of local and spatial factors. J. Limnol..

[5] Petkovski S. (1991). On the presence of the genus *Branchinecta Verril*, 1869 (Crustacea, Anostraca) in Yugoslavia. Hydrobiologia.

[6] Atashbar B., Agh N., Van Stappen G., Beladjal L. (2014). Diversity and distribution patterns of large branchiopods (Crustacea: Branchiopoda) in temporary pools (Iran). J. Arid Environ..

[7] Mura G. (1997). The life history of *Chirocephalus kerkyrensis* Pesta (Crustacea: Anostraca) in temporary water of Circeo National Park (Latium, Italy). Hydrobiologia.

[8] Hulsmans A., Bracke S., Moreau K., Riddoch B.J., De Meester L., Brendoch L. (2006). Dormant egg bank characteristics and hatching pattern of the *Phallocryptus spinosa* (Anostraca) population in the Makgadikgadi Pans (Botswana). Hydrobiologia.

[9] Atashbar A., Agh N., Beladjal L., Jalili R., Mertens J. (2012). Effects of temperature on Survival, Growth, Reproductive and life span characteristics of *Branchinecta orientalis* (Branchiopoda: Anostraca) from Iran. Crustaceana.

[10] Pormehr Yabandeh N., Beladjal L., Agh N., Atashbar B., Van Stappen G. (2017). Mass culture of fairy shrimp *Branchinecta orientalis* (G. O. Sars 1901) (Crustacea: Anostraca) using effluent of rainbow trout *Oncorhynchus mykiss* (Walbaum 1792) ponds. Aquac. Res..

[11] Brett J.R., Hoar W.S., Randall D.J., Brett J.R. (1979). Environmental factors and growth. Fish Physiology: Bioenergetics and Growth.

[12] Biswas A.K., Seoka M., Ueno K., Yong A.S.K., Biswas B.K., Kim Y.S., Takii K., Kumaia H. (2008). Growth performance and physiological responses in striped knifejaw, *Oplegnathus fasciatus*, held under different photoperiods. Aquaculture.

[13] Masoudi Asil S., Fereidouni A.E., Ouraji H., Khalili K.J. (2013). Effects of different light intensities on growth, survival, reproductive and life span characteristics of *Artemia urmiana* Günther 1890. Aquac. Res..

[14] Morales M.I., Barba R.B. (2015). Effects of photoperiod, water levels and sex on the feeding efficiency and weight increment of mudcrabs (*Scylla serrata* Forskall) in a crab-fattening culture system. Int. J. Fish Aquat. Stud..

[15] Hou Z.S., Wen H.S., Li J.F., He F., Li Y., Qi X., Tao Y.X. (2019). Effects of photoperiod and light spectrum on growth performance, digestive enzymes, hepatic biochemistry and peripheral hormones in spotted sea bass (*Lateolabrax maculatus*). Aquaculture.

[16] Moreno-Reyes J., Méndez-Ruiz C.A., Díaz G.X., Meruane J.A., Toledo P.H. (2015). Chemical composition of the freshwater prawn *Cryphiops caementarius* (Molina, 1782) (Decapoda: Palaemonidae) in two populations in northern Chile: Reproductive and environmental considerations. Lat. Am. J. Aquat. Res..

[17] Ueberschär B., Walther B.T., Fyhn H.J. (1993). Measurement of proteolytic enzyme activity: Significance and application in larval fish research. Physiological and Biochemical Aspects of Fish Development.

[18] Herzig A. (1974). Some Population Characteristics of Planktonic Crustaceans in Neusidler Sea. Oecologia.

[19] Hairston N.G., Hansen A.M., Schaffner W.R. (2000). The Effect of Diapause Emergence on the Sysonal Dynamics of a Zooplankton Assemblage. Freshw. Biol..

[20] Vandekerkhove J., Declerck S., Brendonck L., Conde-Porcuna J.M., Jeppesen E., Meester L.D. (2005). Hatching of Cladoceran Resting Eggs: Temperature and Photoperiod. Freshw. Biol..

[21] Sorgeloos P. (1973). First report on the triggering effect of light on the hatching mechanism of *Artemia salina* dry cysts. Mar. Biol..

[22] Takahashi F. (1975). Effect of light on the hatching of eggs in *Triops granarius*. (Notostraca: Triopsidae). Environ. Control Biol..

[23] Alekseev V.R., Hwang J.S., Tseng M.H. (2006). Diapause in aquatic invertebrates: What’s known and what’s next in research and medical application. J. Mar. Sci. Technol..

[24] Wang Z.C., Asem A., Sun S.C. (2017). Coupled effects of photoperiod, temperature and salinity on diapause induction of the parthenogenetic Artemia (Crustacea: Anostraca) from Barkol Lake, China. North–West. J. Zool..

[25] Sorgeloos P., Leger P. (1992). Improved larviculture outputs of marine fish; shrimp and prawn. J. World Aquacult. Soc..

[26] Lim L.C., Soh A., Dhert P., Sorgeloos P. (2001). Production and application of on-grown Artemia in freshwater ornamental fish farm. Aquac. Econ. Manag..

[27] Velu C.S., Munuswamy N. (2007). Composition and nutritional efficacy of adult fairy shrimp *Streptocephalus dichotomus* as live feed. Food Chem..

[28] Sornsupharp S., Dahms H.U., Sanoamuang L. (2013). Nutrient composition of fairy shrimp *Streptocephalus sirindhornae* nauplii as live food and growth performance of giant freshwater prawn postlarvae. Aquac. Nutr..

[29] Sornsupharp B., Lomthaisong K., Dahms H.U., Sanoamuang L. (2015). Effects of dried fairy shrimp *Streptocephalus sirindhornae* meal on pigmentation and carotenoid deposition in flowerhorn cichlid; *Amphilophus citrinellus* (Günther, 1864) × *Cichlasoma trimaculatum* (Günther 1867). Aquac. Res..

[30] Dararat W., Starkweather P.L., Sanoamuang L. (2011). Life history of three fairy shrimps (Branchiopoda: Anostraca) from Thailand. J. Crustac. Biol..

[31] Coutteau P., Brendonck L., Lavens P., Sorgeloos P. (1992). The use of manipulated baker’s yeast as an algal substitute for the laboratory culture of Anostraca. Hydrobiologia.

[32] Chong A.S.C., Hashim R.H., Yang L.C., Ali A.B. (2002). Partial characterization and activities of protease from the digestive tract of discus fish (*Symphysodon aequifasciata*). Aquaculture.

[33] Bernfeld P., Colowick S.P., Kaplan N.O. (1955). α and β amylases. Methods in enzymology.

[34] Iijima N., Tanaka S., Ota Y. (1998). Purification and characterization of bile salt-activated lipase from the hepatopancreas of red sea bream, *Pagrus major*. Fish Physiol. Biochem..

[35] Garcıa-Carreno F.L., Haard N.F. (1993). Characterization of proteinase classes in langostilla (*Pleuroncodes planipes*) and crayfish (*Pacifastacus astacus*) extracts. J. Food Biochem..

[36] Bradford M.M. (1976). A rapid and sensitive method for the estimation of microgram quantities of protein utilizing the principle of protein-dye binding. Anal. Biochem..

[37] Barata C., Varo I., Navarro J.C., Arun S., Porte C. (2005). Antioxidant enzyme activities and lipid peroxidation in the freshwater cladoceran *Daphnia magna* exposed to redox cycling compounds. Comp. Biochem. Physiol..

[38] AOAC (1995). Official Methods of Analysis of the Association of Official Analytical Chemistry.

[39] Folch J., Lees M., Sloane Stanley G. (1975). A simple method for the isolation and purification of total lipides from animal tissues. Inter. J. Biol. Chem..

[40] Ichihara K.I., Shibahara A., Yamamoto K., Nakayama T. (1996). An improved method for rapid analysis of the fatty acids of glycerolipids. Lipids.

[41] Lepage G., Roy C.C. (1984). Improved recovery of fatty acid through direct transesterification without prior extraction or purification. J. Lipid Res..

[42] Lee K.S., Drescher D.G. (1978). Fluorometric amino acid analysis with O-phthaldialdehyde (OPA). Int. J. Biochem..

[43] NRC (2011). Nutrient Requirements of Fish and Shrimp.

[44] Fiogbé E.D., Kestemont P. (1995). An assessment of the protein and amino acid requirements in goldfish (Carassius auratus) larvae. J. Appl. Ichthyol..

[45] Takahashi A., Kasagi S., Murakami N., Furufuji S., Kikuchi S., Mizusawa K., Andoh T. (2016). Chronic effects of light irradiated from LED on the growth performance and endocrine properties of barfin flounder *Verasper moseri*. Gen. Comp. Endocrinol..

[46] Santos Romero R., García Guerrero M., Vega Villasante F., Cortés Jacinto E., Nolasco Soria H. (2017). Effect of photoperiod and temperature on growth and activity of digestive enzymes in juveniles of the long-arm river shrimp *Macrobrachium tenellum* (Smith, 1871) (Caridea: Palaemonidae). J. Crustac. Biol..

[47] Bermudes M., Ritar A.J. (2008). Response of early-stage spiny lobster *Jasus edwardsii* phyllosoma larvae to changes in temperature and photoperiod. Aquaculture.

[48] Dawirs R.R. (1982). Methodological aspects of rearing decapod larvae *Pagurus bernhardus* (Paguridae) and *Carcinus maenas* (Portunidae). Helgol. Meeresunters..

[49] Mikami S., Greenwood J.G. (1997). Influence of light regimes on phyllosomal growth and timing of moulting in *Thenus orientalis* (Lund) (Decapoda: Scyllaridae). Mar. Freshw. Res..

[50] Petit G., Beauchaud M., Attia J., Buisson B. (2013). Food intake and growth of largemouth bass (*Micropterus salmoides*) held under alternated light/dark cycle (12L:12D) or exposed to continuous light. Aquaculture.

[51] Albano M., Panarello G., Di Paola D., Capparucci F., Crupi R., Gugliandolo E., Spanò N., Capillo G., Savoca S. (2021). The Influence of Polystyrene Microspheres Abundance on Development and Feeding Behavior of Artemia salina (Linnaeus, 1758). Appl. Sci..

[52] Dararat W., Lomthaisong K., Sanoamuang L. (2012). Biochemical composition of three species of fairy shrimp (Branchiopoda: Anostraca) from Thailand. J. Crustac. Biol..

[53] Sanoamuang L.O., Saengphan N., Murugan G. (2002). First record of the family Thamnocephalidae (Crustacea: Anostraca) from Southeast Asia and description of a new species of *Branchinella*. Hydrobiologia.

[54] Lancia J.P., Fernandez Gimenez A., Bas C., Spivak E. (2012). Adaptive differences in digestive enzyme activity in the crab *Neohelice granulata* in relation to sex and habitat. J. Crustac. Biol..

[55] Martínez-Alarcón D., Saborowski R., Rojo-Arreola L., García-Carreño F. (2018). Is digestive cathepsin D the rule in decapod crustaceans?. Comp. Biochem. Physiol..

[56] Krylov P.I., Alekseev V.R., Frenkel O.A. (1996). Feeding and digestive activity of cyclopoid copepods in active diapause. Hydrobiologia.

[57] Cronin T.W., Bok M.J., Lin C. (2017). Crustacean larvae—vision in the plankton. Integr. Comp. Biol..

[58] Halliwell B., Gutteridge J.M.C. (1999). Free Radicals in Biology and Medicine.

[59] Pandey S., Parvez S., Sayeed I., Haque R., BinHafeez B., Raisuddin S. (2003). Biomarkers of oxidative stress: A comparative study of river Yamuna fish *Wallago attu* (Bl. & Schn.). Sci. Total Environ..

[60] Tian H.Y., Zhang D.D., Xu C., Wang F., Liu W.B. (2015). Effects of light intensity on growth, immune responses, antioxidant capability and disease resistance of juvenile blunt snout bream *Megalobrama amblycephala*. Fish Shellfish Immunol..

[61] Cañavate J.P., Prieto A., Zerolo R., Sole M., Sarasquete C., Fernandez-Diaz C. (2007). Effects of light intensity and addition of carotene rich *Dunaliella salina* live cells on growth and antioxidant activity of *Solea senegalensis* Kaup (1858) larval and metamorphic stages. J. Fish Biol..

[62] Wang T., Yongzhou C., Zhaopu L., Shaohua Y., Xiaohua L. (2013). Effects of light intensity on growth, immune response, plasma cortisol and fatty acid composition of juvenile *Epinephelus coioides* reared in artificial seawater. Aquaculture.

[63] Jung S.J., Choi Y.J., Kim N.N., Choi J.Y., Kim B.S., Choi C.Y. (2016). Effects of melatonin injection or green-wavelength LED light on the antioxidant system in goldfish (*Carassius auratus*) during thermal stress. Fish Shellfish Immunol..

[64] Wei H., Li H., Xia Y., Liu H., Han D., Zhu X., Yang Y., Jin J., Xie S. (2019). Effects of light intensity on phototaxis, growth, antioxidant and stress of juvenile gibel carp (*Carassius auratus gibelio*). Aquaculture.

[65] Sun X.L., Yang S.Y., Chen C.X., Wang Q.K., Xue-Quan Y.U., Jin-Cheng H.U., Xing K.Z. (2012). Effects of photoperiod on growth and antioxidant indices in half-smooth tongue-sole (*Cynoglossus semilaevis*). Chin. J. Fish..

[66] McGaw I.J., Curtis D.L. (2013). A review of gastric processing in decapod crustaceans. J. Comp. Physiol..

[67] Nemova N.N., Murzina S.A., Lysenko L.A. (2020). Ecological and Biochemical Status of the Atlantic Salmon *Salmo salar* L. and the Brown Trout *Salmo trutta* L. at Early Stages of Development. Biol. Bull. Rev..

[68] Gharibi M.R., Noori A., Agh N., Atashbar B. (2021). Rainbow trout farm effluent as a potential source of feed and medium for mass culture of *Artemia parthenogenetica*. Aquaculture.

[69] Richoux N.B., Ndhlovu R.T. (2014). Temporal shifts in the fatty acid profiles of rocky intertidal invertebrates. Mar. Biol..

[70] Mitra G., Mukhopadhyay P.K., Ayyappan S. (2007). Biochemical composition of zooplankton community grown in freshwater earthen ponds: Nutritional implication in nursery rearing of fish larvae and early juveniles. Aquaculture.

[71] McWhinnie M.A., Kirchenberg R.J., Urbanski R.J., Schwarz J.E. (1972). Crustecdysone mediated changes in crayfish. Am. Zool..

[72] Graney R.L., Giesy J.P. (1986). Seasonal changes in the free amino acid pool of the freshwater Amphipod Gammarus pseudolimnaeus bousfield (Crustacea: Amphipoda). Comp. Biochem. Physiol..

[73] Farhadi A., Harlıoğlu M.M. (2019). Photoperiod affects gamete production, and protein and lipid metabolism in male narrow-clawed Crayfish *Pontastacus leptodactylus* (Eschscholtz, 1823). Anim. Reprod. Sci..

[74] Saejung C., Chaiyarat A., Sanoamuang L.O. (2018). Effects of algae, yeast and photosynthetic bacteria diets on survival and growth performance in the fairy shrimp, *Streptocephalus sirindhornae* (Branchiopoda, Anostraca). Crustaceana.

